# Novel Strategy for Cancer Therapy Proposal Based on Effects and Mechanisms of Targeting Cuproptosis by Polyphenols—A Narrative Review

**DOI:** 10.3390/nu18060917

**Published:** 2026-03-14

**Authors:** Xilong Liu, Mengyao Song, Di Ma, Yiming Pan, Xinqi Liu, Lu Li

**Affiliations:** Key Laboratory of Geriatric Nutrition and Health, Ministry of Education, School of Food and Health, Beijing Technology and Business University, Beijing 100048, China

**Keywords:** polyphenols, cuproptosis, copper homeostasis, oxidative stress, metabolic reprogramming, cancer treatment

## Abstract

As a novel form of cell death, the discovery of cuproptosis presents significant opportunities and challenges for the field of cancer therapy. Notably, polyphenolic compounds have attracted considerable research attention for their ability to induce cuproptosis. These natural compounds not only exhibit marked anti-inflammatory and antioxidant properties, but their polyhydroxy structures also enable effective chelation and transport of copper ions. This provides novel insights into cuproptosis-mediated cancer therapy. Therefore, in this review, we systematically outline copper metabolism, the mechanisms of cuproptosis, and its association with cancer, while providing an in-depth discussion of the effects and mechanisms by which polyphenolic compounds act as copper ionophores to inhibit tumor growth and progression through the induction of cuproptosis. This review indicates the promising potential of polyphenolic compounds in the field of cancer therapy and provides a theoretical basis for therapeutic strategies based on cuproptosis.

## 1. Introduction

Cancer ranks as the second most common cause of death worldwide, only behind cardiovascular diseases, and has become a major burden on public health systems worldwide. According to statistics released by the International Agency for Research on Cancer, approximately 20 million new cancer cases and 9.7 million cancer-related deaths occurred worldwide in 2022 [1]. With the intensification of population aging, shifting lifestyles, and increasing environmental exposure risks, cancer prevention and control may face even more daunting challenges in the future [2]. Against this backdrop, a deep understanding of the mechanisms underlying cancer initiation and progression, particularly its adaptive metabolic alterations, has become pivotal in the search for novel therapeutic strategies.

Metabolic reprogramming is one of the most crucial adaptive alterations in cancer cells. They typically undergo profound metabolic reprogramming, the most prominent feature of which is the preferential generation of energy through aerobic glycolysis even in the presence of ample oxygen—known as the Warburg effect. This phenomenon is often driven by tumor hypoxia, where stabilized hypoxia-inducible factors (HIFs) activate multiple metabolic pathways that enhance glycolysis while suppressing mitochondrial oxidative phosphorylation [3]. Although glycolysis generates ATP at a high rate, its energy conversion efficiency is relatively low. This shift in energy metabolism may interfere with the normal function or efficiency of the Na^+^/K^+^ ATPase, thereby exacerbating the depolarization of the resting membrane potential in cancer cells [4]. This depolarized state blocks the uptake of butyrate by affecting voltage-sensitive transporters such as solute carrier family 5 member 8 (SLC5A8). The resulting decrease in intracellular butyrate levels relieves the inhibition of histone deacetylases (HDACs), thereby reinforcing the Warburg effect [5]. Furthermore, under conditions of metabolic stress, cancer cells further activate AMP-activated protein kinase (AMPK), which downregulates protein synthesis by suppressing the mTOR complex 1 (mTORC1) signaling pathway, thereby enabling adaptation to metabolic pressure under energy-limited conditions [6]. These unique metabolic adaptations enable cancer cells to overcome harsh conditions within the tumor microenvironment, such as hypoxia, acidity, and nutrient deprivation, thereby supporting their rapid proliferation and survival.

Mitochondria, as central hubs of multiple cell death pathways, are capable of integrating signals from diverse stressors to determine cell fate. The direct consequence of metabolic changes in cancer cells is the disruption of mitochondrial reactive oxygen species (ROS) homeostasis. In normal cells, the mitochondrial electron transport chain (primarily at Complexes I and III) continuously generates ROS as byproducts during oxidative phosphorylation. To maintain redox homeostasis, cells precisely regulate ROS levels through the coordinated action of catalase (CAT), superoxide dismutase (SOD), glutathione peroxidases (GPX), and peroxiredoxins (PRDX) [7]. However, in cancer cells, persistent metabolic stress and mitochondrial dysfunction lead to a significant elevation in ROS levels. This elevation exhibits a dual regulatory characteristic in tumor progression: relatively low ROS levels can promote tumor survival and proliferation by modulating oncogenic signaling pathways, whereas excessive ROS accumulation triggers oxidative stress induced cell death, thereby suppressing tumor development [8]. This dual effect reveals the vulnerability of redox balance in cancer cells and underscores the critical role of precisely regulating oxidative stress in cancer therapy.

As an essential trace element, copper plays a central role in key physiological processes, including mitochondrial energy conversion, antioxidant defense, iron homeostasis, and neurotransmitter synthesis (Table 1). Particularly in the regulation of mitochondrial oxidative stress, copper serves as an essential cofactor for various antioxidant enzymes and respiratory chain complexes, playing an indispensable regulatory role. Research indicates that, in comparison to normal cells, tumor cells generally contain elevated levels of copper [9]. This copper accumulation is closely associated with tumor-related inflammation. Inflammatory cytokines such as interleukin-17 (IL-17) promote cellular copper uptake by inducing the expression of the metalloreductase STEAP4. Following copper uptake, the E3 ligase X-linked inhibitor of apoptosis protein (XIAP) is activated, which enhances IL-17-mediated nuclear factor-κB (NF-κB) signaling while inhibiting caspase-3 activity, thereby establishing a positive feedback loop that promotes tumor survival [10]. Recent studies have revealed that excessive accumulation of intracellular copper ions can trigger a novel form of programmed cell death—cuproptosis. This process promotes the aggregation of lipoylated mitochondrial enzymes (particularly components of the pyruvate dehydrogenase complex), disrupts iron–sulfur cluster proteins, and ultimately induces proteotoxic stress, leading to cell death [11]. Furthermore, intratumoral copper has been shown to influence programmed death-ligand 1 (PD-L1) expression in cancer cells, a finding that suggests leveraging copper may enhance the efficacy of immunotherapy [12]. Notably, in contrast to the lack of targeting specificity characteristic of traditional chemotherapy and radiotherapy, cuproptosis, as a form of programmed cell death, may be accompanied by the release of damage-associated molecular patterns (DAMPs), thereby inducing immunogenic cell death (ICD) and activating an anti-tumor immune response [13]. Therefore, leveraging the unique metabolic features of cancer cells—including disrupted redox balance, metabolic reprogramming, and dysregulated copper metabolism—to selectively induce cuproptosis and activate immunogenic cell death pathways has emerged as a highly promising therapeutic strategy.

Natural active substances, with their low toxicity and multi-targeted effects, align with modern pursuits of health therapies. Polyphenols are secondary metabolites widely present in vegetables and fruits, exhibiting significant antioxidant, anti-inflammatory, antiproliferative, and pro-apoptotic activities. They have garnered extensive attention in the fields of cancer chemoprevention and adjuvant therapy [23]. It is noteworthy that the catechol structure abundantly present in polyphenols confers strong Cu^2+^-chelating ability. By forming coordination complexes with polyphenols, Cu^2+^ can achieve transmembrane transport, thereby regulating intracellular copper concentration and subcellular distribution [24]. Furthermore, polyphenols possess significant antioxidant activity and can reduce Cu^2+^ to Cu^+^ via electron transfer pathways, thereby promoting the occurrence of Fenton-like reactions and generating large amounts of hydroxyl radicals, which exacerbates cellular oxidative stress [25]. Based on these properties, polyphenols are regarded as a class of highly promising copper ionophores and cuproptosis sensitizers.

This review systematically summarizes the regulatory mechanisms of copper metabolism and the mechanisms underlying cuproptosis, exploring their application prospects in cancer therapy. By integrating existing research evidence, this review provides an in-depth analysis of the potential pathways through which polyphenolic compounds regulate cuproptosis. Based on a summary of current research limitations, future directions in this field are also discussed, aiming to provide a theoretical foundation and research insights for cancer prevention and treatment strategies based on plant-derived bioactive compounds.

## 2. Cuproptosis: A Copper-Dependent Form of Cell Death

### 2.1. Copper Metabolism

**Figure 1:**
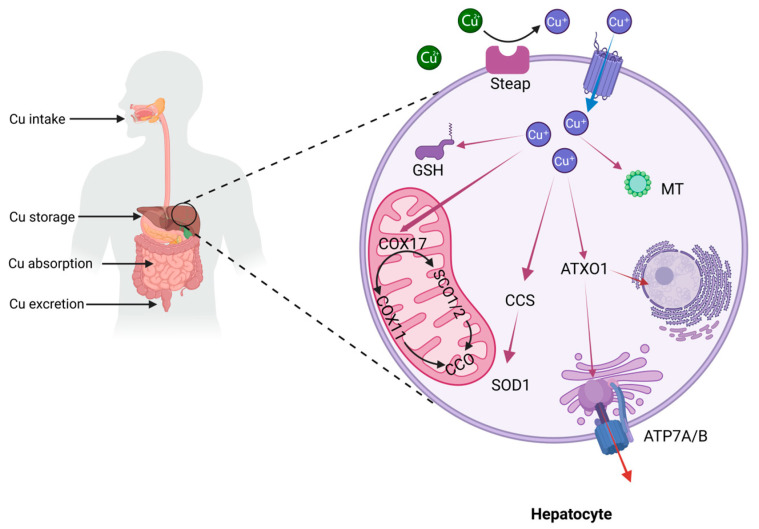
Systemic and cellular copper metabolism. Created in BioRender. [Liu, Xilong]. (2026). https://BioRender.com/cfcjze1 (accessed on 3 March 2026).

Copper is an indispensable trace element for sustaining life activities, performing multiple critical biological functions such as promoting cell proliferation, participating in angiogenesis, supporting respiratory metabolism, and assisting in the elimination of free radicals [26]. However, dysregulation of copper metabolism can lead not only to inherited disorders of copper homeostasis, such as Menkes disease and Wilson disease, but also plays a significant role in the onset and progression of neurodegenerative disorders, anemia, metabolic syndrome, cardiovascular diseases, and various malignancies [27]. Therefore, a comprehensive understanding of the regulatory mechanisms governing copper metabolism is of great scientific and clinical relevance. In mammals, copper metabolism involves systemic and cellular uptake, distribution, storage, and excretion (Figure 1). The human body lacks endogenous copper synthesis pathways and must obtain copper through dietary intake. Nutritional recommendations suggest that adults should consume 0.9 mg of copper daily, primarily derived from copper-rich foods such as shellfish and animal liver [28].

At the systemic level, copper absorption mainly takes place in the small intestine. Copper in the diet primarily exists in the oxidized form Cu^2+^, but its bioavailability is low. Only under low pH conditions does Divalent Metal Transporter 1 (DMT1) exhibit weak transport activity [29,30]. Therefore, ingested Cu^2+^ must first be dissolved by gastric acid and then reduced to Cu^+^ by metal reductases (such as Dcytb1 and Steap2) on the apical membrane surface of intestinal epithelial cells before it can be further absorbed and utilized by the cells [31,32]. Copper is transported into the intestinal lumen via the high-affinity Copper Transporter 1 (CTR1, also known as SLC31A1), which is located on the apical membrane of intestinal epithelial cells [33]. After binding to ligands such as histidine within the intestinal lumen, copper enters the portal venous system. There, it forms complexes with soluble chaperone molecules in the plasma—including albumin, histidine, and ceruloplasmin—and is then delivered to the liver via targeted transport through the bloodstream [34]. The liver plays a key role as the primary organ in copper metabolism within the human body, responsible for multiple physiological functions including copper storage, redistribution, and prevention of copper toxicity [35]. Copper stored in the liver can be re-released into the bloodstream via transmembrane transport mediated by the P-type ATPases ATP7A/B. It then reassociates with soluble copper chaperones and is transported through the circulatory system to target organs such as the heart and brain [36]. To eliminate excess copper, the liver primarily relies on the biliary excretion pathway mediated by ATP7B. Most of the copper is excreted via the biliary system into feces, while the remainder is cleared through glomerular filtration and secretion by cutaneous sweat glands [37,38].

At the cellular level, copper uptake primarily depends on the CTR1, a process regulated dynamically by intracellular and extracellular copper concentrations. Under low-copper conditions, its membrane localization can be maintained by reducing ubiquitination-mediated degradation, thereby enhancing protein stability. Under high-copper conditions, COMM domain-containing protein 1 (COMMD1)-mediated endocytosis leads to the degradation of CTR1, thereby reducing copper uptake efficiency [39]. After entering the cytoplasm, copper rapidly binds to reduced glutathione (GSH) to form low-molecular-weight complexes (Cu-(GSH)_2_), maintaining the concentration of free copper at an extremely low sub-femtomolar level [40]. Various copper chaperone proteins bind to copper through a thiol ligand exchange mechanism and deliver it to specific sites via targeted transport. copper chaperone for superoxide dismutase (CCS) can deliver copper to the active site of SOD1 and promote the dimerization of SOD1 through domain interactions [41]. SOD1 facilitates the conversion of superoxide radicals (O_2_•^−^) into oxygen (O_2_) and hydrogen peroxide (H_2_O_2_), aiding cells in managing oxidative stress. cytochrome c oxidase copper chaperone COX17 (COX17) can transport Cu from the cytoplasm to the inner mitochondrial membrane and deliver it to the cytochrome c oxidase assembly proteins synthesis of cytochrome C oxidase 1 (SCO1) and synthesis of cytochrome C oxidase 2 (SCO2). With the coordinated action of the cytochrome c oxidase assembly protein COX16 and assembly factor cytochrome c oxidase assembly factor 6 (COA6), SCO1 and SCO2 incorporate copper into the mitochondrially encoded cytochrome c oxidase subunit COX2 [42,43]. Additionally, COX17 can also transport and incorporate copper ions into the mitochondrially encoded subunit COX1 through the assembly protein cytochrome c oxidase copper chaperone COX11 (COX11) [44]. The copper centers with redox activity in COX1 and COX2 are essential for the electron transfer within complex IV, which is vital for ATP production. antioxidant 1 copper chaperone (ATOX1) transports copper to the Cu-ATPases ATP7A/B within the Golgi network. ATP7A/B serve as the primary transporters responsible for cellular copper export: ATP7A is predominantly expressed in most non-hepatic tissues, while ATP7B is highly expressed in liver tissue [45]. When intracellular copper levels rise, ATP7A/B relocates from the Golgi apparatus to the plasma membrane, directly exporting copper through a mechanism involving vesicular trafficking and membrane fusion [46]. These transporters ensure the efficient management and clearance of excess copper, thereby safeguarding cells and tissues from copper-induced toxicity.

### 2.2. Mechanism of Cuproptosis

**Figure 2:**
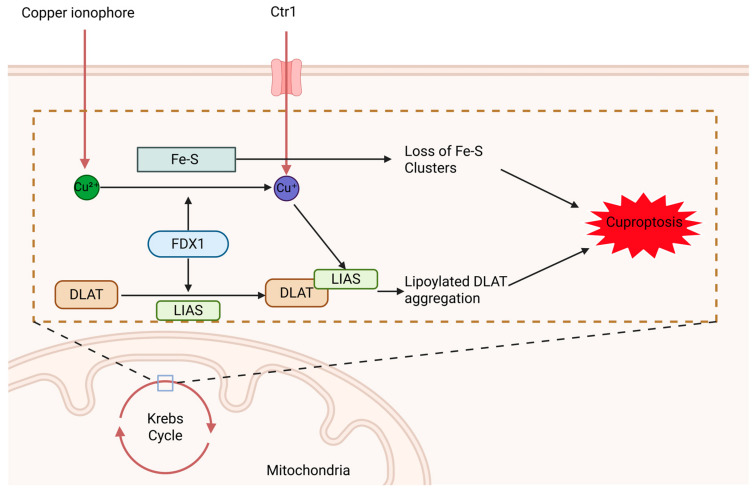
Mechanism of Cuproptosis. Created in BioRender. [Liu, Xilong]. (2026). https://BioRender.com/829un1l (accessed on 3 March 2026).

In normal cells, copper levels are precisely regulated and maintain dynamic homeostasis within the physiological range. When copper homeostasis is disrupted, cells are severely affected. Copper deficiency impairs the function of copper-dependent enzymes, inhibits normal cell proliferation and differentiation, and can potentially cause cell cycle arrest or cell death. In contrast, copper overload generates highly reactive hydroxyl radicals (·OH) via the Fenton reaction, inducing oxidative stress and cellular damage. Notably, when intracellular copper levels exceed a certain threshold, a distinct form of programmed cell death can be triggered [47]. For a period of time, this form of programmed cell death has been intensely studied, yet both its induction conditions and underlying mechanisms have not been fully elucidated. Not until 2019 did Tsvetkov et al. discover that cell death induced by the copper ionophore elesclomol (ES) is highly dependent on its copper-binding ability. ES that completely loses its copper-binding capacity or fails to bind copper ions has no effect on cell viability, thereby providing evidence for a copper-dependent form of cell death [48]. In 2022, the team further revealed that cell death induced by the ES-copper complex does not activate caspase-3, and blocking classical pathways such as apoptosis, necroptosis, or ferroptosis cannot inhibit this process. These findings confirmed a novel form of programmed cell death, which was subsequently termed “cuproptosis.” The TCA cycle is the central metabolic pathway for cellular energy production, in which certain key enzymes require lipoylation to function properly. Lipoylation is a post-translational modification that involves the covalent attachment of fatty acids or lipoic acid molecules to proteins. These lipoylated proteins include dihydrolipoamide S-acetyltransferase (DLAT) in the pyruvate dehydrogenase complex and dihydrolipoamide S-succinyltransferase (DLST) in the α-ketoglutarate dehydrogenase complex [49]. DLAT plays a crucial role in the synthesis of acetyl-CoA. With the involvement of ferredoxin 1 (FDX1), Cu^2+^ are reduced to the more reactive Cu^+^, which then tightly binds to DLAT. This triggers the abnormal aggregation of lipoylated proteins, leading to loss of function and inducing proteotoxic stress [50,51]. Simultaneously, this process leads to the loss of iron–sulfur cluster proteins, impairing mitochondrial function and rendering the cell incapable of sustaining normal metabolism. Ultimately, DLAT aggregation and iron–sulfur cluster depletion act synergistically to drive cell death (Figure 2) [11].

### 2.3. Association Between Cuproptosis and Cancer

Cancer is a complex disease driven by genetic mutations, fundamentally characterized by uncontrolled cell proliferation and immune evasion [52]. Copper ions, as critical effector molecules, are closely linked to the progression of malignant tumors through metabolic dysregulation [53]. Copper ions can directly participate in or activate multiple signaling pathways involved in tumor cell proliferation. For example, copper can bind to receptor tyrosine kinases (RTKs), further promoting the transduction of downstream ERK/MAPK and AKT signaling. Additionally, copper can bind to the histidine residues of PDK1, thereby enhancing phosphoinositide 3-kinase (PI3K)/Akt signal transduction, which collectively strengthens cellular proliferation, migration, and survival capabilities [54,55]. Copper serves as a critical cofactor in intracellular redox reactions and mitochondrial function. It enhances the activity of mitochondrial COX, thereby promoting oxidative phosphorylation and energy supply—processes that are particularly crucial for rapidly dividing tumor cells [56]. Copper can further assist tumor cells in evading immune surveillance, notably through upregulating immune checkpoint proteins such as PD-L1 and modulating inflammatory signaling pathways like NF-κB, thereby enhancing cell proliferation and anti-apoptotic capacity [57]. Furthermore, copper ions play a critical role in tumor angiogenesis. For instance, copper can promote the expression and stabilization of hypoxia-inducible factor-1α (HIF-1α), a key transcription factor that enables tumors to adapt to hypoxic microenvironments. HIF-1α further drives the transcriptional upregulation of vascular endothelial growth factor (VEGF), thereby activating endothelial cell migration and angiogenesis [58]. In addition to its impact on VEGF, copper can also directly bind to other pro-angiogenic factors such as fibroblast growth factor 2 (FGF2), tumor necrosis factor-α (TNF-α), IL-8, and IL-6, or enhance their transcriptional activity by activating the NF-κB signaling pathway, thereby collectively promoting angiogenesis. Compared to normal cells, tumor cells often modulate copper metabolic pathways to acquire more copper, thereby meeting the demands for their rapid growth and proliferation. In individuals with cancers like lung, prostate, breast, gallbladder, gastric, and thyroid cancer, serum copper levels are often significantly elevated. This surplus provides cancer cells with the necessary bioenergy and structural components for their proliferation [57]. Due to their heightened demand for copper, excess copper tends to accumulate more readily in tumor cells. Consequently, in cancer therapy, cuproptosis is regarded as a potential selective killing mechanism for tumor cells. By modulating copper homeostasis or employing copper ionophore-based drugs, tumor cells can be specifically induced to undergo cuproptosis, thereby inhibiting tumor growth while minimizing the impact on normal cells. This offers new possibilities for enhancing therapeutic efficacy while reducing damage to normal cells, as well as overcoming tumor resistance.

The role of cuproptosis across different cancer types remains in the investigational stage, though existing studies have already revealed its potential therapeutic promise in multiple tumor treatments. As shown in Table 2, inducing cuproptosis through strategies such as copper ionophores, natural bioactive compounds, or copper-based nanoplatforms can significantly inhibit cancer cell proliferation and migration, demonstrating promising antitumor prospects. Further investigation into the cellular effects of cuproptosis and strategies to enhance cellular sensitivity to it will be of significant value.

## 3. Polyphenols: A Class of Structurally Diverse Natural Copper Chelators

**Figure 3:**
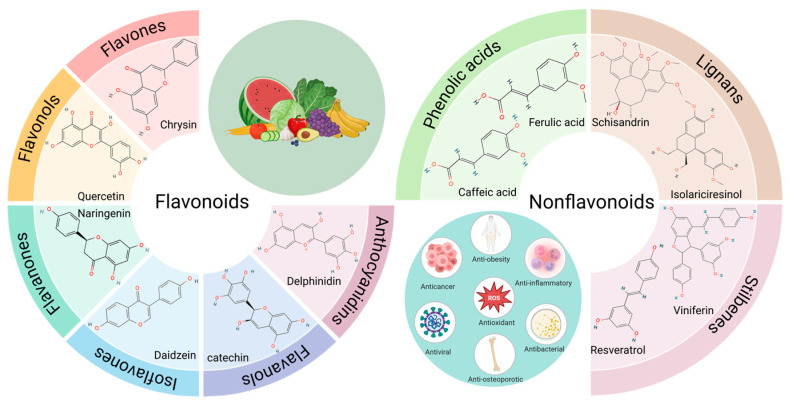
Classification of Polyphenols. Created in BioRender. [Liu, Xilong]. (2026). https://BioRender.com/ks1kyx0 (accessed on 3 March 2026).

Polyphenolic compounds are phytochemicals characterized by a benzene ring structure bearing two or more phenolic hydroxyl groups, which are plant secondary metabolites. They are widely distributed in the roots, stems, leaves, and fruits of higher plants, exhibiting significant antioxidant, anti-inflammatory, antiproliferative, and pro-apoptotic activities. They represent one of the most structurally diverse classes of natural products known, with the broadest spectrum of biological activities [70]. Based on their chemical structures and origins, they are primarily classified into two major categories: flavonoids and non-flavonoids (Figure 3). Flavonoids predominantly exist in glycosidic forms and encompass a highly diverse range of subclasses, including flavones, flavonols, flavanones, flavanols, isoflavones, and anthocyanidins. Non-flavonoid compounds mainly comprise phenolic acids, stilbenes, and lignans, among others [71].

**Figure 4:**
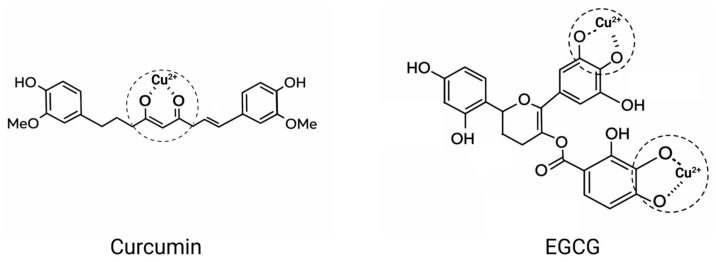
Schematic diagram illustrating the interaction between polyphenols and copper ions.

The molecular structure of polyphenols is rich in characteristic functional groups, such as catechol and galloyl moieties, which confer upon them a strong ability to chelate metal ions. They can form stable five or six-membered chelate rings with copper ions (Figure 4) [72]. From a chemical structural perspective, when phenolic hydroxyl groups approach copper ions, the oxygen atoms on the phenolic hydroxyls possess lone pair electrons, while the copper ions have vacant orbitals. The lone pair electrons enter the vacant orbitals of the copper ions, thereby forming stable coordinate bonds and achieving chelation with the copper ions [73]. The β-diketone moiety in curcumin exhibits keto-enol tautomerism similar to that of acetylacetone, and its enol form can coordinate with copper ions to form a stable six-membered chelate ring [74]. EGCG can form two stable five-membered chelate rings with copper ions, with copper coordinated by the hydroxyl groups on the B-ring and the galloyl moiety, thereby inhibiting ROS generation [75]. Quercetin possesses three potential binding sites for copper ions. A six-membered chelate ring can be formed at the β-ketophenolic moiety (5-hydroxy-4-keto), while a five-membered ring is formed at the α-keto-enol site (3-hydroxy-4-keto). The third metal-binding site involves the catechol group, which exhibits both antioxidant and copper-chelating activities. However, there is controversy surrounding the β-ketophenolate and α-ketoenolate binding sites of flavonoids [72]. The binding properties between polyphenols and copper ions confer upon them a unique ability to regulate intracellular copper concentration and control its subcellular distribution, highlighting the significant potential of polyphenols in promoting cuproptosis by modulating copper accumulation. The binding strength between polyphenols and copper ions is influenced by pH. In acidic environments, copper ions more readily form stable complexes with polyphenols, whereas under alkaline conditions, the stability of these complexes weakens, allowing copper ions to be released [76]. This controllable stability is highly significant in biomedical applications. In drug delivery systems, it enables precise control over the release of copper ions from Cu-polyphenol complexes. Furthermore, copper-polyphenol complexes can specifically accumulate in tumor regions and precisely target the mitochondria of cancer cells.

Metal-phenolic nanocomposites constructed from polyphenols have been successfully employed to induce cuproptosis (Table 3), highlighting the substantial potential of natural polyphenols in regulating this process. It is worth emphasizing that the role of polyphenols in this context extends beyond merely acting as copper ionophores; they also participate in and regulate the occurrence of cuproptosis through multiple mechanisms.

## 4. Polyphenols Sensitize Cuproptosis: Multidimensional Mechanisms from Homeostasis Regulation and Metabolic Remodeling to Synergistic Lethality

### 4.1. Targeted Copper Management: Laying the Material Foundation for Cuproptosis

Cuproptosis is essentially a form of programmed cell death triggered by excessive copper ions. Its occurrence depends on the accumulation of intracellular free copper ions exceeding a critical threshold. Therefore, disrupting copper homeostasis and promoting intracellular copper accumulation constitute the primary material basis for inducing cuproptosis.

The intracellular copper concentration in tumor cells is primarily regulated by a network of copper transport proteins, and the dysregulation of this system is considered a critical factor in inducing cuproptosis. Studies have shown that polyphenolic compounds can directly target the copper transport system, enhancing copper uptake and reducing copper efflux. CTR1 is a key protein for copper uptake in tumor cells and serves as the primary pathway for maintaining copper homeostasis. EGCG enhances CTR1 mRNA and protein expression in ovarian cancer cells and in xenograft mouse models, while also inhibiting the rapid degradation of CTR1 induced by cisplatin (cDDP). EGCG has also been shown to upregulate the transcription and protein expression of CTR1 in lung cancer cells. This effect can be further amplified through the ROS-mediated ERK1/2–lncRNA NEAT1 signaling axis, thereby enhancing cellular copper uptake [86,87]. The expression of CTR1 in mammals is subject to regulation by the specificity protein 1 (Sp1) transcription factor [88]. Curcumin can enhance the expression of CTR1 by upregulating and modulating the Cu–Sp1–CTR1-positive feedback regulatory loop [89]. Similarly, luteolin has also been demonstrated to upregulate Sp1 through activation of the PI3K/Akt signaling pathway [90]. Furthermore, polyphenols not only promote copper uptake but also inhibit copper efflux by downregulating the expression of ATP7A and ATP7B, key transporters responsible for cellular copper export. Curcumin has been shown to suppress the expression of ATOX1 and ATP7A in NSCLC cells [91]. Naringin, EGCG, and soybean isoflavones can inhibit the upregulation of ATP7A caused by excessive copper concentration in MCF-10A cell lines [92,93]. Recent studies have found that EGCG suppresses the expression of MTF1 in HCC cells, thereby attenuating its transcriptional activation of ATP7B. This restricts copper efflux, promotes copper accumulation within tumors, and significantly enhances cellular sensitivity to cuproptosis [94].

Beyond transport processes, the “availability” of intracellular free copper is also tightly constrained by ligand buffering systems. GSH plays a crucial role in cellular antioxidant defense, detoxification processes, and the maintenance of redox homeostasis. Simultaneously, as an endogenous copper chelator, it effectively regulates intracellular copper ion levels, keeping them within the physiological range and preventing the toxic effects caused by excessive copper accumulation [95]. GSH inhibits the occurrence of cuproptosis. Depletion of GSH can significantly increase the proportion of intracellular free copper, thereby providing a prerequisite for triggering cuproptosis [96]. Studies have shown that various polyphenols demonstrate the capacity to inhibit GSH synthesis. For example, curcumin suppresses GSH synthesis in HSCs by inhibiting the expression of methionine adenosyltransferase (MAT) via p38 MAPK phosphorylation and by interfering with *C*-Jun *N*-terminal kinase (JNK) signaling [97,98]. Resveratrol can also inhibit hepatic GSH synthesis by suppressing MAT [99]. Compounds such as EGCG can inhibit cystathionine β-synthase (CBS) activity, blocking the conversion of methionine to cysteine and thereby limiting GSH synthesis [100,101]. Polyphenols not only inhibit GSH synthesis; studies have also found that flavonoids such as apigenin and naringenin can act on the ABC transporter multidrug resistance-associated protein 1 (MRP1), thereby blocking GSH transport and leading to GSH depletion [102]. Polyphenols can effectively promote intracellular copper ion accumulation by modulating copper transport proteins and inducing GSH depletion, thereby demonstrating significant potential in regulating copper homeostasis.

### 4.2. Metabolic Reprogramming: Creating a Cuproptosis-Sensitive Cellular Environment

Cuproptosis primarily occurs in cells reliant on oxidative phosphorylation. However, to meet the energy demands of rapid proliferation, malignant tumor cells often reprogram their energy metabolism networks, with the Warburg effect (i.e., aerobic glycolysis) being the most prominent feature [103]. The Warburg effect is manifested by a significant increase in the expression of key glycolytic enzymes such as phosphofructokinase (PFK), pyruvate kinase (PK), hexokinase (HK), and lactate dehydrogenase A (LDHA). Enhanced aerobic glycolysis reduces glucose utilization efficiency. To satisfy the increased energy requirements for cell proliferation, tumor cells enhance the expression of glucose transporter 1 (GLUT1), a key protein involved in glucose uptake [104]. Under the synergistic action and excessive activation of these enzymes, a large amount of pyruvate is converted into lactate, thereby reducing acetyl-CoA synthesis. This in turn suppresses the mitochondrial oxidative phosphorylation process, significantly lowering the sensitivity of tumor cells to cuproptosis. Studies have shown that inhibiting the activity of key enzymes in aerobic glycolysis can reduce glycolytic flux, thereby effectively potentiating the cuproptosis response [105]. Many polyphenols can inhibit the Warburg effect by directly or indirectly modulating the activity of glycolysis-related enzymes. Quercetin inhibits the expression levels of GLUT1, PKM2, and LDHA through the Akt-mTOR pathway, thereby reducing glucose uptake and suppressing lactate production, which ultimately decreases cellular glycolysis [106]. Another study also demonstrated that quercetin reduces the expression of HK-2 in HCC cells, inhibits HK2-dependent glycolysis, and thereby suppresses cell proliferation [107]. EGCG significantly inhibits the expression of phosphofructokinase (PFK) and monocarboxylate transporter 4 (MCT4), reduces lactate production in cancer-associated fibroblasts (CAFs), suppresses aerobic glycolysis activity, and thereby reverses the Warburg effect [108]. Curcumin (diferuloylmethane) downregulates PKM2 through inhibition of the mTOR-HIF1α axis, thereby reducing energy metabolism in various cancer cells—including those from lung, breast, and cervical cancers—and attenuating the Warburg effect [109]. Treatment of E705 cells with polyphenols from G. officinalis extract reduces ATP content, suppresses glycolytic function, and specifically downregulates markers of the Warburg effect [110]. Studies have found that resveratrol enhances PDH activity by activating AMPK, promotes oxidative phosphorylation, and reverses the Warburg phenotype [111]. Resveratrol reduces glucose uptake and lactate production by inhibiting the mTOR/PKM2 axis, thereby suppressing glycolysis and tumor metabolism [112]. Interestingly, the combined treatment of curcumin, quercetin, and resveratrol shows a significant synergistic effect. Compared to single agents, this combination more effectively reduces the activity of key metabolic enzymes. These enzymes are involved in glycolysis, respiration, one-carbon metabolism, glutaminolysis, and fatty acid synthesis. This effect has been observed in human neuroblastoma cell lines and mesenchymal stem cells, demonstrating that multi-target inhibition of metabolic reprogramming can be achieved [113]. Therefore, in future applications, it would be valuable to further explore combination strategies involving multiple polyphenols, as this approach holds promise for achieving more pronounced metabolic regulatory effects.

### 4.3. Intensifying Oxidation: Driving the Positive Feedback Loop in Cuproptosis Execution

**Figure 5:**
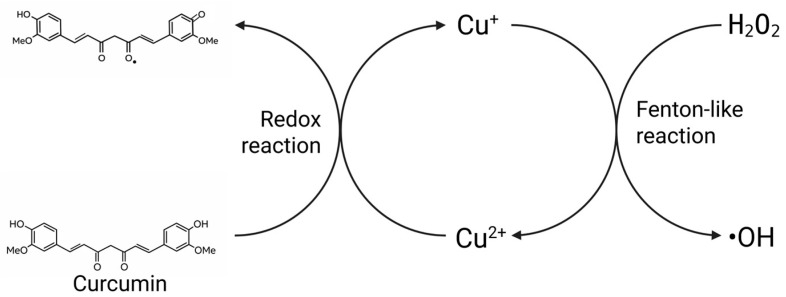
Polyphenols interact with excess copper ions to promote the Fenton-like reaction.

While cuproptosis does not rely on oxidative stress for its occurrence, oxidative stress and cuproptosis are closely intertwined. Copper stress not only induces cuproptosis in tumor cells but also generates abundant ROS through Fenton-like reactions, thereby triggering oxidative stress and leading to cellular oxidative damage [114]. Additionally, intracellular copper ions, when chelated with GSH, can further exacerbate cellular oxidative damage. While oxidative stress enhances the expression of CTR1 and ATP7A induced by copper stress, promoting intracellular copper accumulation, it further upregulates the expression of FDX1 and TCA-related proteins, thereby facilitating the occurrence of cuproptosis [115]. The positive feedback mechanism formed between cuproptosis and oxidative stress plays a key regulatory role in its execution process. Therefore, inducing oxidative stress in tumor cells can further intensify the cuproptosis process, thereby enhancing antitumor efficacy. The role of polyphenols in regulating oxidative stress has been extensively studied. In normal cells, polyphenols exhibit significant antioxidant activity. Their phenolic hydroxyl groups can directly react with ROS and RNS, scavenging free radicals and mitigating the level of intracellular oxidative stress [116]. However, in the presence of transition metal ions such as copper and iron, the antioxidant activity of polyphenols can convert to pro-oxidant activity [117]. For example, when ellagic acid (EA) is used in combination with Cu^2+^, it leads to the generation of ROS, significantly increasing the formation of 8-oxo-dG in calf thymus DNA and resulting in oxidative DNA damage [118]. In the presence of transition metal ions such as copper, resveratrol can exhibit pro-oxidant properties, leading to oxidative breakage of cellular DNA [119]. Gallic acid (GA) can induce death in A549 lung cancer cells by increasing ROS production and promoting GSH depletion [120]. When the intracellular Cu^2+^ concentration is elevated, it interacts with EGCG, promoting its conversion from an antioxidant to a pro-oxidant state, thereby inducing cellular oxidative stress [121]. This occurs because Cu^2+^ first chelates with the ortho-dihydroxyl groups of polyphenols and is reduced to Cu^+^. Subsequently, Cu^+^ further reduces O_2_ to superoxide anion (O_2_•^−^), which then undergoes dismutation to produce H_2_O_2_. Ultimately, Cu^+^ catalyzes the conversion of H_2_O_2_ into hydroxyl radicals (•OH) via a Fenton-like reaction, generating large amounts of ROS [122]. In this process, polyphenols act as electron donors, continuously reducing Cu^2+^, thereby sustaining and amplifying the copper-dependent Fenton-like reaction (Figure 5). This exacerbates oxidative stress and generates more toxic Cu^+^. Cu^+^ is not merely a reduced form of copper; rather, it serves as a key effector in the execution of cuproptosis. This ion can form strong complexes with DLAT, leading to its abnormal aggregation. Consequently, it disrupts the stability and synthesis of Fe–S cluster proteins, ultimately accelerating the onset of cuproptosis. The regulation of oxidative stress by polyphenols is dependent on intracellular copper ion concentration. This characteristic enables them to induce cuproptosis in tumor cells while protecting normal cells from oxidative damage. Such a context-dependent dual regulatory mechanism demonstrates excellent selectivity in cuproptosis-targeted cancer therapy, allowing specific elimination of tumor cells and offering a novel perspective for developing precision anticancer strategies.

### 4.4. Synergistic Lethality: Converging Multimodal Cell Death Pathways for Therapeutic Synergy

Programmed cell death plays a central role in maintaining tissue homeostasis and inhibiting tumor progression. Tumor cells often gain survival advantages by evading single death pathways. Therefore, the simultaneous activation of multiple cell death mechanisms is considered an important strategy to enhance therapeutic efficacy. Recent studies have shown that polyphenolic compounds, in addition to inducing cuproptosis by regulating copper homeostasis, can also synergistically activate apoptosis, ferroptosis, and autophagic cell death, thereby forming a synergistic anti-tumor effect with cuproptosis at multiple levels. Studies have found that various polyphenols can regulate GPX4, iron homeostasis, and lipid peroxidation to drive the ferroptosis signaling pathway, thereby inhibiting tumor growth [123]. For example, Selaginellaceae induces ferroptosis in MCF-7 cells by inhibiting the ubiquitination and proteasomal degradation of voltage dependent anion channel 2 (VDAC2), triggering mitochondrial lipid peroxidation, and promoting ROS production [124]. Typanomycin (TYP) can trigger autophagy in AML cells by promoting the activation of AMPK signaling, leading to ferritin degradation and ROS accumulation, thereby inducing ferroptosis [125]. Cuproptosis and ferroptosis have overlapping points in metal homeostasis disruption, mitochondrial dysfunction, and redox imbalance. GSH, as a cofactor of GPX4, helps reduce lipid peroxidation and suppress ferroptosis [126]. Copper ions can bind to GSH, depleting GSH reserves and thereby promoting lipid peroxidation and inducing ferroptosis. The ferroptosis process is accompanied by substantial ROS production, which further exacerbates mitochondrial oxidative stress, consequently forming a mutually reinforcing positive feedback loop between “ferroptosis and cuproptosis” [127].

Copper stress can trigger substantial production of ROS through catalyzing Fenton-like reactions, inducing cellular oxidative stress. This process also collaboratively activates the tumor suppressor p53, thereby promoting apoptosis [128]. Latest reviews indicate that a variety of polyphenols (such as apigenin, quercetin, curcumin, resveratrol, and EGCG) can cooperatively upregulate the expression, protein stability, and post-translational modifications (such as phosphorylation, acetylation, and methylation) of p53, thereby enhancing p53-induced apoptosis [129]. Activation of p53 can upregulate the expression of FDXR, which facilitates the transfer of electrons from NADPH to FDX1. This process further promotes the biosynthesis of Fe-S clusters and drives the progression of cuproptosis [130].

Copper can trigger autophagy by activating AMPK or inhibiting the AKT pathway, which in turn suppresses mTOR activity, as well as by directly binding to ULK1/2. Additionally, copper-induced ROS can further enhance the autophagic process through activation of the Nrf2 pathway [131]. Research has demonstrated that polyphenols, including quercetin, resveratrol, and curcumin, promote autophagy in cancer cells by regulating the AMPK/mTOR, Beclin 1, and LC3 signaling pathways [132,133,134]. Furthermore, polyphenol-mediated downregulation of ATP7B reduces mTOR activity, which in turn promotes the nuclear translocation of TFEB and enhances cellular autophagy [135]. Polyphenols and copper ions can synergistically induce autophagy in cells. Under conditions of elevated copper levels, the activated autophagy may exacerbate cellular stress, thereby promoting cell death [136]. Polyphenols and copper ions can synergistically induce programmed cell death, thereby further promoting cuproptosis.

This multi-pathway synergistic mechanism is expected to reduce the ability of tumor cells to evade any single death modality, thereby providing a new theoretical basis for combined anti-cancer therapeutic strategies that utilize polyphenols to target cuproptosis.

## 5. Polyphenols in Cuproptosis: Limitations and Translational Hurdles

Although polyphenolic compounds have shown considerable promise as potential regulators of cuproptosis and possess inherent advantages as natural products, current research remains largely confined to the preclinical stage, with evidence primarily derived from in vitro cell experiments and animal models. Their clinical efficacy and safety have yet to be rigorously validated, and the translation from bench to bedside still faces significant challenges.

The pharmacokinetic properties and in vivo metabolic processes of polyphenolic compounds are critical factors limiting their clinical translation. The absorption of polyphenols primarily occurs in the small intestine. However, most polyphenols are easily hydrolyzed in the digestive tract and rapidly metabolized in the intestinal wall or liver. Coupled with the barrier function of the intestinal lining, these factors result in their generally low bioavailability [137]. Only a small number of polyphenols can enter the bloodstream. Due to their lack of specific targeting ability, these trace components can only be distributed non-specifically throughout the body via the bloodstream, unable to accumulate effectively at target sites. Even if some polyphenols accumulate to a certain extent in organs such as the liver, kidneys, or brain, their actual concentration in target tissues is often insufficient to reach therapeutically effective thresholds, largely due to their poor lipophilicity and low aqueous solubility [138]. The metabolic transformation of polyphenols in vivo occurs primarily in the intestine and liver. Through the action of phase I and phase II metabolizing enzymes—such as UDP-glucuronosyltransferases (UGTs), sulfotransferases, and methyltransferases—polyphenols are converted into glucuronidated, sulfated, or methylated conjugates. These conjugates exhibit significantly enhanced water solubility but generally possess markedly reduced biological activity and therapeutic potential compared to their parent compounds [139]. Taking resveratrol as an example, it is rapidly transformed in vivo primarily through glucuronidation and sulfation pathways in the intestine and liver, resulting in the formation of conjugates such as resveratrol 3-O-glucuronide and resveratrol-3-sulfate. Consequently, the parent compound is detected at very low levels in circulation [140]. The excretion of polyphenols and their metabolites occurs primarily via the urinary and biliary routes. Unabsorbed fractions may be further metabolized by the gut microbiota and subsequently eliminated in the feces [141]. Due to the rapid metabolism and high clearance rate of most polyphenols in vivo, their half-lives are generally short, resulting in limited duration of action. Consequently, when used as monotherapy, their therapeutic effects are often less pronounced than those of conventional chemotherapeutic agents or targeted drugs.

The potential systemic toxicity of polyphenols should also be considered. Studies have shown that polyphenols can exhibit vastly different biological effects depending on dosage, cell type, and microenvironmental conditions. Inappropriate use may not only fail to achieve the desired therapeutic effects but may also lead to cellular damage and even systemic toxicity. Polyphenols may exhibit opposite biological effects depending on the dose—a phenomenon known as dose-dependent biphasic effects. For example, resveratrol exhibits cytotoxic effects on normal cells at high concentrations and may damage non-target cells by inducing apoptosis or oxidative stress. In animal models, high-dose resveratrol has been shown to induce pathological changes such as renal tubular dilation and acute nephritis in mice, as well as increase serum urea nitrogen and liver enzyme levels, suggesting potential toxic effects on vital organs including the liver and kidneys [142]. Polyphenols exhibit considerable variation in their effects across different cell types. For example, in various cancer cell models, EGCG significantly inhibits tumor growth through mechanisms such as cell cycle regulation and apoptosis induction. In contrast, its effects are generally milder in normal cells [143]. Furthermore, the tumor microenvironment can significantly influence the therapeutic efficacy of polyphenols. Transition metal ions present in the tumor microenvironment, such as copper and iron, can alter the redox behavior of polyphenols, shifting them from antioxidants to pro-oxidants. Specifically, polyphenols interact with transition metal ions through Fenton-like reactions, promoting the generation of ROS, thereby exacerbating intracellular oxidative stress and inducing DNA damage [144].

The stability of complexes formed between polyphenols and copper ions represents another critical limitation for their clinical application. In low-pH environments such as the stomach, these complexes generally exhibit higher stability. However, in neutral or alkaline environments, copper ions may undergo dissociation from the coordination structure or even be reduced to the unstable Cu^+^ form, leading to complex disassembly and loss of efficacy [145]. This pH-dependent characteristic poses certain challenges for the absorption and distribution of polyphenols. In the stomach, the low pH favors the stability of copper complexes, suggesting that drug release within the gastric environment may be relatively stable and effective. However, in more neutral or alkaline environments such as the intestine, copper ions may dissociate from polyphenols or be converted into less stable forms, thereby reducing their efficacy at these sites.

## 6. Targeting Cuproptosis with Polyphenols: Prospects and Outlook

The discovery of cuproptosis has revealed a profound connection between tumor metabolism and metal homeostasis, opening new avenues for cancer therapy. Although the current strategy of targeting cuproptosis with polyphenols faces numerous challenges, concerted efforts in fundamental research, preclinical studies, and the optimization of drug delivery systems are expected to overcome these obstacles and advance the clinical translation of this approach in cancer treatment.

Future research should further deepen the exploration of the molecular mechanisms by which polyphenols regulate cuproptosis. Although this review has systematically outlined the potential mechanisms involved, whether polyphenols can directly or indirectly target core regulatory molecules of cuproptosis, such as FDX1, LIAS, and DLAT, requires systematic validation through molecular docking techniques combined with in vitro and in vivo experiments. Furthermore, recent studies have shown that polyphenols can influence epigenetic processes, including DNA methylation, histone modifications, and non-coding RNA expression [146]. Whether these epigenetic changes are involved in regulating the expression of cuproptosis-related genes represents an equally urgent scientific question warranting in-depth investigation.

To address the inherent limitations of polyphenols—such as low bioavailability, poor selectivity, and insufficient stability—future research should focus on optimization at both the molecular structure and drug delivery system levels. At the molecular level, medicinal chemistry approaches can be employed to modify the polyphenol backbone, improving their pharmacokinetic properties while retaining copper-chelating activity. The introduction of specific functional groups, such as halogens, methyl groups, or phosphate groups, can enhance metabolic stability, increase water solubility, or confer targeting capabilities, thereby providing directions for the development of novel polyphenol derivatives [147]. At the delivery level, the application of nanotechnology provides powerful tools for enhancing the tumor-targeting and bioavailability of polyphenols. Various carriers, including liposomes, polymeric nanoparticles, metal–organic frameworks, and exosomes, enable passive or active targeted delivery of polyphenols [148]. Of particular interest are smart delivery systems with pH-responsive, enzyme-responsive, or multi-stimuli-responsive capabilities. These systems can regulate drug release based on the characteristics of the tumor microenvironment, thereby improving therapeutic efficacy while reducing systemic toxicity.

In light of the limited efficacy of polyphenols as monotherapy, combination therapy will become the primary direction for their clinical application. Numerous studies have demonstrated that polyphenols can enhance the antitumor effects of chemotherapeutic agents such as cisplatin, doxorubicin, and paclitaxel. The underlying mechanisms involve inhibiting drug efflux, reversing drug resistance, and synergistically inducing cell death [149]. The characteristics of cuproptosis—namely, the release of DAMPs and the induction of ICD—provide a theoretical basis for combining polyphenols with immune checkpoint inhibitors. Polyphenol-induced cuproptosis may reshape the tumor immune microenvironment and enhance antitumor immune responses. This direction warrants in-depth preclinical investigation, including exploration of its potential in combination with PD-1/PD-L1 antibodies, CTLA-4 antibodies, and other immunotherapeutic agents. Furthermore, complex crosstalk exists among various programmed cell death pathways, including ferroptosis, pyroptosis, and autophagy [150]. Whether polyphenols can simultaneously regulate multiple death pathways, as well as the synergistic or antagonistic relationships between different modes of cell death, represents an important topic for future investigation.

Safety concerns regarding the clinical translation of polyphenol-cuproptosis strategies must also be addressed. The absence of specific biomarkers for monitoring cuproptosis in clinical settings hinders real-time detection and therapeutic assessment. Additionally, the long-term safety of polyphenol–copper complexes, especially their potential impact on copper-dependent enzyme activities, remains to be established. Systematic studies—including acute/chronic toxicity, reproductive toxicity, genotoxicity, and drug interaction evaluations—are essential to support their safe clinical application.

## 7. Conclusions

This review demonstrates that polyphenolic compounds can act as effective promoters of cuproptosis through multi-layered synergistic mechanisms. The complexes formed by polyphenols and copper selectively accumulate in tumor regions and target cancer cell mitochondria, increasing local copper concentrations while minimizing adverse effects on normal tissues. Building on this, polyphenols effectively induce cuproptosis in tumor cells through multiple synergistic pathways, including the regulation of copper transporters, reprogramming of tumor cell energy metabolism, and generation of reactive oxygen species via Fenton-like reactions. Furthermore, polyphenols can simultaneously activate other forms of programmed cell death, such as apoptosis, autophagy, and ferroptosis, creating a multifaceted antitumor effect that further enhances their therapeutic potential.

However, the mechanisms described above are currently based largely on preclinical evidence. Whether they can be precisely and reproducibly translated to complex clinical settings, and how the context-dependent effects of polyphenols—such as dosage and microenvironment—influence their therapeutic efficacy, remain central challenges and open questions. Nevertheless, polyphenol-based strategies targeting cuproptosis, as an emerging direction in cancer therapy, have demonstrated unique theoretical value and application potential. In the future, with deeper insights into their molecular mechanisms, innovations in targeted delivery technologies, and optimization of combination therapeutic strategies, this innovative approach holds promise to advance from bench to bedside, offering novel and more promising treatment options.

## Figures and Tables

**Table 1:** Functions and Main Tissue Distribution of Major Human Copper-Dependent Enzymes.

Enzyme	Function	Copper Cofactor	Main Tissue Distribution	References
Cytochrome c oxidase (COX)	Electron transfer in mitochondrial respiration	Cu^2+^	Ubiquitous (high in heart, liver, brain, skeletal muscle)	[14]
Superoxide dismutase 1 (SOD1)	Antioxidant	(Cu^2+^/Zn^2+^)	Cytoplasm of all tissues; high in liver, brain, erythrocytes	[15]
Ceruloplasmin	Ferroxidase; copper transport	Cu^2+^	Liver	[16]
Dopamine β-hydroxylase (DBH)	Catecholamine biosynthesis	Cu^+^/Cu^2+^	Adrenal medulla, noradrenergic neurons of sympathetic nervous system	[17]
Tyrosinase	Melanin biosynthesis	Cu^+^/Cu^2+^	Melanocytes (skin, hair, eyes), retinal pigment epithelium	[18]
Lysyl oxidase (LOX)	Oxidative deamination	Cu^2+^	Connective tissues (heart, lung, skin, aorta, bone)	[19]
Hephaestin	Ferroxidase (intestinal iron export)	Cu^2+^	Small intestine (basolateral membrane of enterocytes)	[20]
Peptidylglycine α-amidating monooxygenase (PAM)	Peptide hormone amidation (*C*-terminal α-amidation)	Cu^+^/Cu^2+^	Neuroendocrine tissues, pituitary gland, brain	[21]
Superoxide dismutase 3 (SOD3, EC-SOD)	Antioxidant (extracellular superoxide dismutation)	(Cu^2+^/Zn^2+^)	Extracellular matrix of lung, blood vessels, heart, kidney	[22]

**Table 2:** Summary of Compounds Inducing Cuproptosis in Cancer Models.

Compounds	Cancer	Model	Mechanism	Reference
Eupalinolide B	Pancreatic cancer	MiaPaCa-2, PANC-1, PL-45, HPNE	Increases ROS levels, disrupts copper homeostasis, and enhances elesclomol cytotoxicity.	[59]
Triptolide	Cervical cancer	HeLa, SiHa	Modulates XIAP/COMMD1/ATP7A/B axis, disrupts copper homeostasis, and induces cuproptosis.	[60]
ZnPT	Triple-negative breast cancer	MDA-MB-231	Disrupts copper homeostasis and promotes DLAT oligomerization, inducing cuproptosis.	[61]
CAPSH	Breast cancer	4T1, Male Balb/c nude mice	Synergistic copper release and siRNA-mediated copper homeostasis disruption elevate intracellular copper levels, inducing cuproptosis.	[62]
NP@ESCu	Bladder cancer	BIU-87, T24, MB49, Male Balb/c nude mice	Reprograms tumor microenvironment, binds αPD-L1, and efficiently delivers copper to induce cuproptosis.	[63]
ZCProP	Breast cancer	4T1, 4T1 mouse breast tumor model	Promotes direct binding to lipoylated TCA cycle proteins, inducing cuproptosis.	[64]
GOx@ [Cu(tz)]	Breast cancer	MCF-7, MCF-10, 5637, SV-HUC-1, Male Balb/c nude mice	Reduces glucose and GSH levels, promotes aggregation of lipoylated mitochondrial proteins, and sensitizes cells to cuproptosis.	[65]
Lipo-Ele@CuO_2_	Synergistic Cancer	LLC, Male Balb/c nude mice	Delivers copper ions, depletes GSH, and sensitizes cells to cuproptosis	[66]
CuP/Er	Colon cancer	4T1, MC38	Depletes GSH, enhances Warburg effect, and exacerbates cuproptosis.	[67]
METTL16	Gastric cancer	METTL16-WT, K229R, K229E	Upregulates FDX1 expression, thereby inducing cuproptosis.	[68]
Cu-VT	Bladder cancer	MB49, C57BL/6 nude mice	Transports copper ions, promotes GSH depletion and ROS production, and induces cuproptosis.	[69]

**Table 3:** Applications and Effects of Polyphenols in Polyphenol–Copper Complex-Induced Cuproptosis.

Polyphenols	Polyphenol-Copper Complex	Effect	Reference
Gallic acid (GA)	Cu-GA NPs	Coordinates with copper ions, depletes intracellular GSH, and disrupts redox homeostasis, thereby sensitizing cells to cuproptosis.	[77]
Shikonin (SK)	ACH NPs	Binds to copper via coordination and enhances oxidative stress, contributing to cuproptosis induction.	[78]
Curcumin	Bm–Cur–Cu_3_@RBCm	Forms copper-coordinated complexes, depletes GSH, and downregulates ATP7B expression, promoting cuproptosis.	[79]
Piceatanol (Pic)	Cu-Pic/HA NPs	Coordinates with copper, inhibits polyamine synthesis and uptake, depletes intracellular polyamines, and induces mitochondrial dysfunction, leading to cuproptosis. Coordinates with copper, inhibits polyamine synthesis and uptake, depletes intracellular polyamines, and induces mitochondrial dysfunction, leading to cuproptosis.	[80]
Hyaluronic acid (HA)	MgO_2_-CuO_2_@HA NC	Forms coordination complexes with Mg^2+^ and Cu^2+^, triggers Fenton-like reactions to generate •OH, and depletes GSH, facilitating cuproptosis.	[81]
Tannic acid (TA)	CuO_2_@G5-BS/TF	Coordinates with copper and inhibits carbonic anhydrase IX (CA IX) activity, contributing to cuproptosis sensitization.	[82]
Tannic acid (TA)	TAF-CuET	Coordinates with copper to catalyze Fenton-like reactions, generating abundant ROS, depleting GSH, inducing mitochondrial damage, disrupting ATP production, and suppressing ATP7A/ATP7B expression, thereby promoting cuproptosis.	[83]
Tannic acid (TA)	T-T@Cu	Enhances the enhanced permeability and retention (EPR) effect via copper coordination, enabling effective tumor accumulation in 4T1 tumor-bearing mice and facilitating cuproptosis.	[84]
Gallic acid (GA)	CuFeGA MPNs	Coordinates with Cu^2+^ and Fe^2+^, depletes GSH, and promotes Fenton reactions with H_2_O_2_, generating ROS and inducing lipid peroxidation (LPO), thereby sensitizing cuproptosis.	[77]
Chlorogenic acid (ChA)	ChA-Cu NPs	Forms coordination complexes with copper, depletes GSH, and disrupts redox homeostasis, contributing to cuproptosis induction.	[85]

## Data Availability

The original contributions presented in the study are included in the article, and further inquiries can be directed at the corresponding author.
